# The Relation of Medical Editors to the Medical Profession in the United States

**Published:** 1876-08

**Authors:** A. N. Bell

**Affiliations:** President


THE
### SOUTHERN MEDICAL RECORD.

VOL. VI. AUGUST, 1876. NO. 8

### Original Communications.

### THE RELATION OF MEDICAL EDITORS TO THE MEDICAL PROFESSION IN THE UNITED STATES.

ANNUAL ADDRESS BEFORE THE AMERICAN MEDICAL EDITORS' ASSOCIATION, PHILADELPHIA, JUNE 5, 1876.

By A. N. BELL, M.D., President.

Medicine as defined by the dictionary is that science which has for its object "the cure of disease and the preservation of health,"* a definition founded in error and perpetuated by pretenders. As thus defined it has been the stock in trade for a horde of mere prescribers for symptoms in all ages, and the foundation of the vulgar acceptation of medicine: that it consists of experimental knowledge only, and that its benefits may be measured by the number of alleged cures that have been effected—by the number of persons who may be duped into the belief that because they have taken certain prescriptions and survive, they are examples of recovery from disease. But deception in medicine is not exclusively confined to the pretensions of charlatans. It is quite as often due to insufficiently educated practitioners, clothed with all the credentials of the

*Dunglison.

profession—and thus rendered capable of redoubled mischief—who impose upon the credulity of their patients, and bring disgrace upon the profession of which they are accredited members.

There is no need in this connection to sketch the history of medicine. It is sufficient to state, medicine existed when history began; that it is no less true now than it was under the observation of Homer, that "One doctor is worth a host of other men to cut out arrows and apply simple dressings." But the time has long since gone by, when the sphere of doctors was confined within such narrow bounds.

Mankind seems to have always been sensible of the importance of the knowledge of remedies to assuage pain; and the discovery of means for the prevention and cure of disease has in all ages been deemed too important a matter not to be recorded. From the earliest ages the simplest information of this kind has been preserved, and modified or extended, according to the accumulated experience of the most enlighted portion of every community. By this means medical literature has accumulated in such abundance, that he who undertakes to fathom it, is in danger of being lost in the vast labyrinth of conflicting theories and dogmas; insomuch, that were it possible to reduce the merely valuable portion of medical libraries to the dimensions of well-written magazine literature, all could be contained on very few shelves—and the world would be the gainer by condemning all the rest to the waste-basket.

Let it not be supposed, however, that in passing this judgment little estimate is to be placed on the works of the fathers in medicine. It has been well said by some one, that in the progress of scientific truth the ancients are in truth our juniors; those who come first, our ancestors, are the young people, having had the least experience, and to it we have added the experience of many centuries; and, therefore, as far as experience goes, are wiser and more capable of correct conclusions than our fathers were. Of the living votaries of medicine, therefore, at any given period, it may be truthfully said, to the oldest is to be accorded the most experience and therefore the most wisdom, but in the progress of science through generations of men, directly the reverse of this proposition is true.

But young, ignorant and inexperienced as the fathers of medicine were, we should not on that account disparage them. The wonder is not that they knew so little, but that they knew so much. Erroneous, it is true, have many of their conclusions been proven to be, and the same fate doubtless awaits many of our conclusions. We, too, in the light of future ages will be regarded as tyros; and lest we be careful of our record, the mazes of infinitesima and the gibberish of clairvoyance will be looked back upon by future generations, as no less a part of medicine in our age, than were the hidden mysteries of alchemy, astrology, and witchcraft which we find pinned on to the coat-skirts of our fathers.

It has been well said by a distinguished writer, "That man is not the discoverer of any art who first says the thing; but he who says it so long, and so loud, and so clearly, that he compels mankind to hear him—the man who is so deeply impressed with the importance of the discovery that he will take no denial, but at the risk of fortune and fame rushes through all opposition, and is determined that what he thinks he has discovered shall not perish for want of a fair trial."*

It is doubtless true, to a certain extent, of individuals in all the avenues of life, that to impose upon the world is to make their fortune, and to tell it a truth which it did not know before is to make their ruin equally sure. But however true of individuals this axiom may be, it is by no means true of science. Pare, Harvey, Jenner, and many others might be cited as examples of men who suffered severely for the cause of truth, who were sure of their knowledge, and persisted in promulgating their discoveries to the ruin of their temporal prospects, but the benefits which they conferred on science will live forever. Other examples might be cited of benefits conferred on medicine by men who were not physicians. Indeed, some there have been who conferred benefits by their open opposition and ridicule. Such an one was Pliny, the naturalist, who, while he derided medi-

*Sydney Smith's Essays, Langman's Edition, p. 523.

cine, by his researches in natural history contributed greatly to its resources.

The discovery of anæsthesia, the greatest discovery of modern times, if not, indeed, of all time, as a resource of medicine, was not made by a physician. And in the discovery of anæsthesia, by the way, we have an illustration of the principle just quoted in regard to what constitutes discovery. For, granting that Wells did all that his friends claim for him, that on the 10th day of December, 1844, on witnessing some experiments made with nitrous oxide, he discovered that a person who had received severe bruises while under its influence was insensible of them, and that upon this he drew the inference which he subsequently put in practice to the extent of drawing some teeth without pain, he nevertheless failed in an effort to impress his discovery on the practical attention of surgeons, and apparently abandoned the effort. Morton, on the contrary, whether the idea was original with him or not, had such faith in the anæsthetic effect of ether, which he used instead of the nitrous oxide with which Wells had failed, that he not only challenged its use in a surgical operation, but he published it in every accessible way possible to him, and at his hands the medical profession received it, no matter who first thought of it.

Many remedies known to be directly curative of certain diseases, such as cinchona and its salts, sulphur, mercury, cod-liver oil, lemon-juice, etc., are the result of empirical observations. And so, too, it was with regard to the great discoveries of anæsthesia and vaccination.

By accident or experiment it is discovered that a certain substance is of use in a particular disorder. The same remedy is subsequently and repeatedly administered in like condition, and upon a number of snch data an empirical system is established. This kind of practice is in accord with the vulgar acceptation of the practice of medicine, and requires but little knowledge. Those who accept it as the sum of medical knowledge stand in the same relation to scientific medicine as the ancients. And by a precipitation of judgment common to the unenlightened, they assert remedies of whose properties they know nothing for diseases of which they are equally ignorant, and call themselves physicians. But however limited the knowledge and extensive the danger of such practitioners, the physio-pathological phenomena in the application of remedies empirically are valuable, because their utility is made known by therapeutical proof. Improvements in medicine have frequently been derived from most humble sources, or seized upon under the most fortuitous circumstances.

Medicine is no exception to the general truth, that the most perfect science is that which reconciles and brings together the greatest number of facts, that can come within the sphere of the subject of it. And medical progress, as a whole, is to be regarded as the result of the successful labors of many individuals. While a few persons may be identified with certain improvements of their time, it is none the less true, as a general rule, that such persons deserve credit for only a part of the progress made under their names. Indeed, silent workers often render the most efficient service, confirming or refuting the published accounts of the few; and every single ste,) in the direction of prevention and cure is progress.

Founded as this Association is, upon a common allegiance to the medical profession, and a clear appreciation of the advantages common to combined effort in the cause of science, its field of labor is obvious: to cultivate and disseminate scientific truth; to boldly expose without fear or favor the abuses which threaten the well-being of the profession; to rouse the attention of the public to a sense of the danger of ignorant and unprincipled persons, who either assume for themselves, or have conferred upon them, the titles of "doctor," "pharmacist" and "apothecary" —at the eminent hazard of the lives of all persons who are so unfortuate as to become the subjects of their care.

Emancipated alike from the colleges and the enfranchisements of mouldy "authorities," on becoming editors, we enter a field of labor which admits of no middle ground. Embracing modern research and cultivation, as vehicles for truth and ordeals for what is unsettled, it is expected of us to perceive the truth and reflect the light. Editors cannot afford to be content with any place below the front rank, and their duty is to maintain its lines with unflinching integrity; to be constantly on the alert, to scrutinise, and to assualt or espouse, as the case may be, the wrong or the right in every cause for its inherent qualities; but always with care and politeness, lest sharp criticism be mistaken for prejudice, or favor for flattery. Nothing but that which one really performs can be an honor to him, and what he claims or takes from another more than he ought, deserves exposure.

In one respect at least the medical practitioner and the medical editor occupy common ground: neither is safe in his practice or his reputation who is afraid to face the case in hand in all its bearings; and in both, he is most to be relied on who is best fitted to intelligently comprehend tne emergency before him, and to deduce from the signs or evidences present, the probable tenor of coming results. "If false facts," remarks Lord Bacon, "be once set on foot, what, through neglect of examination, the countenance of antiquity, and the use made of them in discourse, they are scarce ever retracted." Wary, however, as we should ever be for the integrity and honor of our calling, it behooves us at all times to be careful of snap judgments. To no class of men in the world is time for examination, reflection and knowledge more necessary than to editors, and to no editors so much as medical editors; for in no other profession is the field of innovation so broad, or fraught with the necessity of more careful watching than medicine. To it the spirit of innovation is literally abroad, from within and without, and so it has been from the beginning—more "doctors" than of any other occupation.

Joubert, a sprightly writer of three centuries ago, after having shown the popular presumption of so many persons assuming to act as physicians without the requisite knowledge, narrates the following adventure: "It is said that the Duke of Ferrara, Alphonso d'Este, at one time proposed in a familiar way the question, 'In what calling are most men engaged?' One said 'shoemakers;' another 'tailors;' a third 'carpenters, masons, pettifoggers and laborers.' Gonelle, his famous buffoon, said there were more physicians than any other class of men, and made a bet with the duke, who denied it, that he would prove it in twenty-four hours. The next morning Gonelle left his lodgings wearing a great night-cap and a cravat tied around his chin, then a hat over all, and his mantle drawn up over his shoulders.

"In this attire he took a route through a populous street leading to the palace of his master. The first person he met asked him what was the matter? He replied that he had a raging toothache. 'Ha, my friend,' said the stranger, 'I know the best remedy in the world against it, and told it to him. Gonelle inscribed his name on his tablet, pretending that he was writing down his recipe.

"A step further on he found two or three together, who all asked the same question, and each one gave him a remedy. He inscribed their names as the first. And thus he pursued his course, very carefully, to the end of the street, not meeting a single person who did not offer him a recipe, and different from the rest, each one assuring him that his remedy was established, certain and nearly infallible. He wrote down the names of all. Approaching the lower court of the palace, he found himself surrounded with gentlemen (for they all knew him), who, after having learned his affliction, compelled him to take their recipes also, which each one assured him was the best in the world. He thanked them all and wrote down their names. When he entered the chamber of the duke, his excellency cried out, 'Eh! what is the matter, Gonelle?'

"He replied very piteously and complainingly: 'The toothache; the worse that ever was.' To which his excellency replied, 'Ha! Gonelle, I know a thing which will drive off the pain at once, without touching the tooth; Mr. Antonio Musa Brussavolo has never employed a better one. Do so and so, and immediately you will be healed.' Gonelle suddenly threw off his head-dress and his attire, crying out, 'And you, also, my lord, are a physician! look at my list, and see how many others I have found between my lodgings and your palace. Here are nearly two hundred, and I have but passed through one street; I will engage to find ten thousand in the city, if I go everywhere. Find me as many persons in any other business.'"*

*Erreurs popularies par Laurent Joubert, 1587.

Since Joubert's time matters in this regard have not improved. But from the unbounded conceit and credulity of human nature, which is confined to no particular age or generation, much useful information has been deduced. Many useful remedies have been first brought to the knowledge of physicians through the desperate efforts of individuals to relieve pain, or the hazardous experiments of fools and knaves, without any regard whatever to the philosophy of medicine; and a poor physician is he surely who refuses or neglects the use of remedies because they have not come down to him through the restricted channel of medical ethics, or turns his back upon those who venture to fly the beaten track in search of new truths. To collate and apply the truth from all sources is pre-eminently the sphere of scientific medicine. Charlatanism consists much more in deception and dishonesty than in ignorance, and it has been fostered not a little by the mischievous aim of medicine as declared in its ordinary definition, already mentioned.

It seems to be an inherent vice of human society, that there should always exist in every community, a considerable number of persons so constituted as to become the willing subjects of imposition. For the most part this portion of society consists of two classes of persons. First, of those whose minds are so wrapped up in their own pursuits as to have no time or taste for other knowledge, and therefore accept on trust the specious pretensions of charlatans; and second, those who consider themselves the progressive class of society, who are constantly in search of new things, and are readily captivated by anything which is out of the usual order of intelligence—anything which in its nature partakes of the marvelous. To these a few may be added—disappointed invalids with incurable diseases—who are willing to try anything which promises relief; more deserving of pity, certainly, these, for the additional afflictions which they suffer, than either of the two chief classes named, which in the main constitute the field of labor for all those who, both in their practice and their faith, regard the science of medicine as a mere trade in physic. It would be better for scientific medicine, if physicians would more generally recognize this condition of human society as unavoidable. Quacks of all kinds are necessary to them, and they sooner or later reap the reward of their folly. But one dishonest pretender for the time being will make more headway among such people than a dozen honest and well educated physicians. And if, as in the too common society action of physicians, any such pretender, or it may be a union of pretenders who constitute a sect, in either case, is publicly ostracized, such acts always redound to the benefit of the quack. He can well afford means which honorable men eschew, to persuade his patrons of his superiority—as the chief cause of unpopularity among the "old-school doctors." They like to be persecuted —they can turn it to account. The best way of treating quacks —and those who patronize them—is with the utmost degree of polite circumspection; but during the while, physicians should never neglect to avail themselves of the opportunities they afford for getting information, by post mortem examinations.

The mere "cure of disease" should no longer obtain as a primary object of medicine in either faith or practice. But instead the admirable object, so well comprehended by the elder Bigelow more than twenty years ago, which may be defined: *A knowledge of diseases and their causes, and the art of preventing them; aud curing them when curable.** Let this be made the standard of professional competency, by proper restrictions and tests, and one important step, at least, will have been taken toward the acquirements necessary for a sound professional education, and the exclusion of all those who boast the use of a remedy for every symptom, or one remedy for all symptoms, in the face of their daily history, which gives the lie to all snch assertions.

But let it not be for a single moment supposed, that we consider the course of medical instruction, as ordinarily pursued in this country, calculated to sustain this precept, or to hinder the progress of quackery. On the contrary, we think it eminently calculated to promote quackery. From about fifty catalogues and announcements of Medical Colleges before me at the time of this writing, the following extract was selected from the one

*Nature in Disease, by Jacob Bigelow, M.D., p. 69.

representing the largest number of students, as a fair representation of the requirements for graduation by colleges of the highest standing:

"Three years pupilage after eighteen years of age, with a *regular physician in good standing*, inclusive of the time of attendance upon medical lectures; attendance on two full courses of lectures, the last being in this college; certificates of at least one course of practical anatomy, or dissections; proper testimonials of character; an acceptable thesis composed by and in the handwriting of the candidate; and a satisfactory examination in each of the seven departments of instruction, viz: practice of medicine, surgery, obstetrics, materia medica, physiology, anatomy and chemistry. The examinations upon practice of medicine and surgery include pathological anatomy, ophthalmology, and diseases of the skin. Two full courses of lectures are absolutely required, *and no period of practice is taken as an equivalent for one course.* The candidate must be twenty-one years of age. The three years recognized are considered as ending at the close of the winter session. In this provision, the three years date from the time of graduation, and practice before graduation is not counted. The winter session begins October 1st and ends about 1st of March. A "matriculating examination" on preparatory education is advertised, which "is optional with the student, and will be given by the faculty to all who desire it. * * * It is necessary for those only who expect to present their tickets or diplomas for recognition in Great Britain."

The faculty for the winter course of this college, including professors of special departments, numbers *nineteen;* and *fourteen* assistants. Besides the duty of hearing so many lectures, numerous hospitals and public institutions have to be visited, dissection practiced, and the use of various instruments and mechanical appliances learned.

That such a process of cramming, can result in the acquirement of practical knowledge, is inconsistent with human intelligence. At best, it conveys but a mere smattering of the subjects gone over—not even laying a good foundation for subsequent study.

Impressed with an idea of a "regular course" merely, such graduates are, as a rule, only competent to rival quackery—more likely to acquire its arts, than to overthrow them.

While this association does well not to assert itself in any respect a reformatory body, it should, nevertheless, be more alive to the general trend of medical instruction, and show less confidence than it has hitherto shown in the promised reform of medical colleges by professors and teachers. *Their* reform consists in the progressive disgrace of the profession, by the bestowment of its honors upon all who are competent to pay the college fees.

Modern medicine has expanded beyond the bounds of any individual comprehension, and subdivision has become no less essential for its cultivation than its practice. The specialties of a few years ago have become departments; and, that the attempt on the part of professors and teachers, to continue to explain the whole of the sciences now embraced in medicine to students within the brief space of time commonly allowed, has resulted in the utter failure and degradation of the profession, is in no respect surprising. And this undertaking is the more to be wondered at, when we consider that many of the professors and teachers themselves make little or no pretension to practical knowledge outside of their special departments, both in and out of college. And yet we find a dozen or more of these learned gentlemen hammering away at the young intellect at the rate of six or eight hours a day, and every night, for two sessions of four months each, and then *passing* them as having acquired knowledge enough to practice medicine in *all* its branches!

The absurdity of the system is only exceeded by its danger, and the more from the circumstance that with few exceptions no standard of preparatory education is required.

The appreciation of our short-comings in this respect is by no means limited to Great Britain, as the advertisement quoted would lead students to suppose, but well nigh universal.

In a recent paper on "European Ideas of Medical Education in the United States," by Thomas P. Corbally, M.D., of Brooklyn, after noticing the villainous practice of selling American medical diplomas in Europe, and the probable action of the German Government for its prohibition, we have the following *resume* from the report of Valcourt, in 1868, to the Minister of Public Instruction on Medical Institutions in the United States: "The time required for medical studies is nominally three years, but is in reality reduced to two; for the student must spend the third year in following the civil practice of some physician in good standing, and must bring proof by a certificate from his preceptor that he has complied with this formality. Besides this, the year is limited to the winter session of four months. During the course of his studies, the student is not subjected to any examination. To be admitted to the degree of doctor he must be twenty-one years of age, and transmit to the dean of the faculty a thesis written by himself. If the thesis is considered satisfactory, the student is examined by each professor separately; the examination is not public, and is often given during a *tete-a-tete*, at the residence of the professor. When all the students have been interrogated in this way, the faculty assemble and confer on all those that have received less than three black balls the diploma of doctor of medicine. The number of candidates rejected is insignificant."*

From the report of the Commissioner of Education of the United States, we learn that the total number of schools of medicine, dentistry and pharmacy reported to that office for 1874, was 99; the number of instructors, 1,121; students, 9,095. Of these 9,095 students reported, 733 only are shown to have received a degree in letters or science (omitting any account of 59 out of the whole 99 institutions which failed to make any report on this point;) and five expressly state that in their classes there are no college graduates. The total number of volumes in the libraries of these schools, as far as appears from their reports, is only 66,611; from 44 there was no response to questions under this head, and one distinctly reported that it had none.

While the total benefactions to education in the United States for the year were, so far as ascertained, $6,053,304, these

*Proceedings Medical Society of the county of Kings, Brooklyn, Vol. I., p. 6.

several schools, which so directly affect human life, received for the year only $308,466, and a total income of $24,219 from permanent funds, being almost entirely dependent upon their tuition fees, which amount to $520,593.

In view of this showing, and the humiliating advertisement of one of our chief colleges—that the standard of qualification for admission to our ranks is below that which will entitle the holder to recognition abroad—we may well be alarmed at the prospective future of medicine in the United States. This is no time to take part in the conflict of sects for ascendancy in certain universities. It is the time for action on the part of the American Medical Association, by which a standard of professional qualification may be fixed independent of the colleges; a standard to which they—the colleges—shall be required to conform, or else denied the privileges of the association. The time for appointing committees "to report at the next session," or for longer dependence on the promises of the colleges, is passed. The danger of all such delays is upon us, and the present is the time for action. The vague and indeterminate generalities which have served no good purpose in the past, are not likely to promise any better results for the future, and if the standard of medical education in the United States is to be raised at all, it must be raised by its highest tribunal—the American Medical Association.

But medical editors have no need to wait for ceremony in this regard. Their liberty and their duty is to expose existing abuses, and, if possible, render them so odious as to make their reform a necessity. Our medical colleges must be made to feel that their period of unexampled prosperity, under existing regulations, shall no longer continue to be a period of peace. And, if I may be permitted, in conclusion, to apply one of the wholesomest axioms of sanitary science to the most important of all subjects which now concerns the medical profession in the United States—the low standard of professional education—my proposition is, from this time forth until it is reformed, to treat it as an intolerable nuisance. By universal assent, *the fittest time for the removal of a nuisance is the very earliest day practicable*

*after its existence has been made known.* Whoever opposes the removal of it on that day, will be sure to oppose it, if he dare, on every other day.

